# Development of a Simple Formula for Identifying Reduced Fat-Free Mass in Health Screening Participants

**DOI:** 10.3390/jcm15166431

**Published:** 2026-08-20

**Authors:** Hiroki Nishikawa, Hyeongki Park, Raiki Yoshimura, Shingo Iwami, Akira Asai, Soo Ki Kim, Akira Fukuda, Sachiyo Yoshio

**Affiliations:** 1 The Second Department of Internal Medicine, Osaka Medical and Pharmaceutical University, Takatsuki 569-8686, Osaka, Japan; hiroki.nishikawa@ompu.ac.jp (H.N.);; 2 School of Biomedical Convergence Engineering, Pusan National University, Yangsan 50612, Republic of Korea; 3 Interdisciplinary Biology Laboratory (iBLab), Division of Natural Science, Graduate School of Science, Nagoya University, Nagoya 464-8601, Aichi, Japan; 4 Department of Gastroenterology, Kobe Asahi Hospital, Kobe 653-8501, Hyogo, Japan; 5 Health Science Clinic, Osaka Medical and Pharmaceutical University, Takatsuki 569-8686, Osaka, Japan; 6 Department of Human Immunology and Translational Research, National Institute of Global Health and Medicine, Japan Institute for Health Security, Shinjukuku 162-0052, Tokyo, Japan

**Keywords:** sarcopenia, low muscle mass, fat-free mass index, screening model, prediction model

## Abstract

**Background/Objectives**: To develop and validate a simple prediction model for identifying individuals with low fat-free mass index (FFMI) using routinely available clinical variables. **Methods**: A total of 8736 individuals were included (mean age = 54.2 years). Among men, 1674 (40.1%) had a low FFMI (<18 kg/m^2^), while among women, 2160 (47.3%) had a low FFMI (<15 kg/m^2^). Candidate predictors were preprocessed to reduce redundancy and multicollinearity. Repeated LASSO logistic regression and XGBoost-based SHAP analysis were used to identify robust predictors, and sex-specific logistic regression models were developed using the final selected variables. Model performance was evaluated using 500 repeated 8:2 train-test splits, calibration analysis, risk stratification, and decision curve analysis (DCA). **Results**: BMI and estimated GFR (eGFR) were consistently selected as key predictors and were used to construct the final sex-specific models. The BMI-eGFR model showed excellent discrimination. Calibration plots demonstrated good agreement between predicted probabilities and observed event rates. Mean held-out sensitivities and specificities were 0.861/0.875 in men and 0.869/0.805 in women, respectively. DCA showed that the model provided a higher net benefit than treat-all or treat-none strategies over most of the evaluated threshold range of 0.05–0.50. **Conclusions**: Our model for predicting low FFMI may be helpful when direct muscle mass measurement is not readily available.

## 1. Introduction

In general, physical function tends to decline with age [1]. Skeletal muscle can be an organ that regulates the body’s homeostasis [2]. Since the medical term “sarcopenia” was included in the ICD-10 in 2016, research on sarcopenia has made significant progress [3,4]. In Asian Working Group for Sarcopenia (AWGS) 2025, the diagnostic algorithm for sarcopenia is based solely on reduced muscle mass and reduced muscle strength, while physical function is used as an outcome measure [5]. Another major change in AWGS2025 compared to AWGS2019 is the introduction of new diagnostic criteria for sarcopenia not only for the cohort aged 65 and older but also for the cohort aged 64 and younger [5,6]. With this change, we have reached a major turning point: the concept of “sarcopenia,” which was previously associated with older adults, is now being applied to the assessment of sarcopenia in younger individuals as well.

Numerous previous studies have reported that decreased muscle mass can be an adverse prognostic factor [7,8,9,10]. However, in clinical practice, particularly when measuring muscle mass using CT or dual energy X-ray absorptiometry, it is time-consuming and labor-intensive to measure muscle mass in all patients. Therefore, it seems more practical to identify patients at risk of muscle mass loss and measure their muscle mass. The AWGS guidelines recommend screening for cases of reduced muscle mass using the SARC-F or the finger-circle test [5,6]. However, while the SARC-F questionnaire and the finger-circle test are easy-to-use assessment tools, they have the drawback of lacking quantitative data. A simple and highly quantitative formula for predicting muscle mass is expected to be beneficial in clinical practice. Fat-free mass (kg) is calculated by subtracting body fat from total body weight. Skeletal muscle accounts for about 50% of fat-free mass [11]. Fat-free mass index (FFMI, kg/m^2^) is a well-established marker for muscle mass assessment and can be closely linked to metabolic health [11].

In this study, we used FFMI as a representative indicator of muscle mass and aimed to develop a simple and clinically applicable prediction model using routinely available clinical variables obtained from health screening participants. To identify robust and interpretable predictors, we combined repeated LASSO-based variable selection with XGBoost-based SHAP analysis, incorporating both statistical stability and machine learning-based feature importance. Based on these analyses, we developed a simplified risk model using only body mass index (BMI) and estimated glomerular filtration rate (eGFR), and evaluated its predictive performance, calibration, risk stratification ability, and clinical utility.

A simple and quantitative screening tool for identifying low FFMI may facilitate the early identification of individuals at risk of decreased muscle mass in real-world clinical settings. Furthermore, such a model may contribute to more efficient allocation of muscle mass measurements and effectively support large-scale population screening strategies in both younger and older adults. The proposed model was intended as a preliminary screening rule rather than a substitute for direct muscle-mass measurement or a definitive diagnostic tool for sarcopenia.

## 2. Patients and Methods

### 2.1. Description of Cohort and Study Design

**Figure 1:**
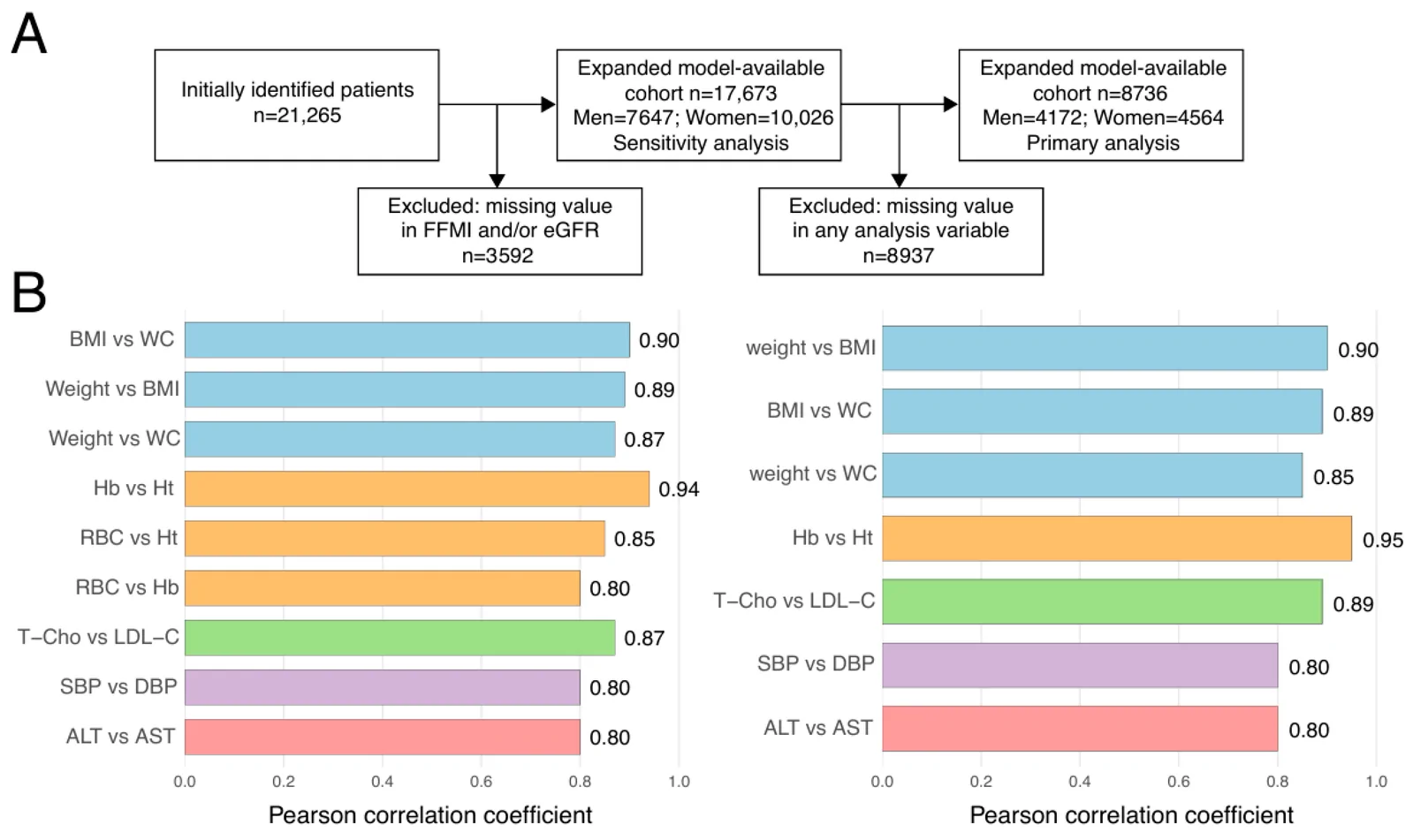
**Cohort selection and highly correlated clinical variable pairs:** (**A**) Flow diagram of the complete-case cohort selection. Of 21,265 initially identified participants, 8736 were included in the final analysis cohort after excluding those with missing values. (**B**) Highly correlated clinical variable pairs in men and women subgroups. Pearson correlation coefficients were calculated among clinical variables, and variable pairs with absolute Pearson correlation coefficients |r| ≥ 0.80 are shown. Bar colors indicate clusters of highly correlated variable pairs corresponding to similar clinical domains. Values at the end of the bars indicate Pearson correlation coefficients. BMI, body mass index; WC, waist circumference; Hb, hemoglobin; Ht, hematocrit; RBC, red blood cell; SBP, systolic blood pressure; DBP, diastolic blood pressure; ALT, alanine aminotransferase; AST, aspartate aminotransferase.

Clinical data used in this study were collected from participants who visited Health Science Clinic, Japan, between February 2022 and June 2024. A total of 21,265 participants were initially identified. Participants with missing values in any of the variables used for the analysis were excluded, resulting in a final cohort of 8736 individuals. Therefore, all analyses were conducted using a complete-case cohort without missing data (Figure 1A).

The mean age of the final cohort was 54.2 years (standard deviation [SD], 11.8 years), ranging from 20 to 92 years. The cohort comprised 4172 men and 4564 women (Figure 1A). Because the primary objective of this study was to investigate reduced FFMI, all subsequent analyses were performed separately for men and women to account for sex-specific physiological differences. Basic demographic information, including BMI and age, with blood-test-derived clinical variables, were also collected. Because these variables are commonly available in clinical practice, the proposed approach has high potential for practical implementation. Detailed baseline characteristics of the study population are presented in Table 1.

Variable-specific missingness in the initial cohort and a comparison of participants included in and excluded from the complete-case analysis are presented in Table S1 and Table S2, respectively. The specific reasons for missingness could not be determined from the retrospective dataset, and the missing data were therefore not assumed to be completely at random. No additional range-based exclusion, transformation, or winsorization of implausible values was applied to the analytical dataset.

### 2.2. Assessment of Muscle Mass and Definition of Low FFMI

FFM (kg) was measured by bioelectrical impedance analysis (BIA) (TANITA, body composition analyzer with automatic height meter, DC-270A-N, Tokyo, Japan). The BIA was performed by a well-trained technician in the morning and on an empty stomach, while the participant was standing quietly in light clothing. FFMI was calculated as FFM (kg) divided by the square of height (m^2^). Kawakami et al. reported that using Japanese large sample (n = 1313, aged 40–87 years), the suggested FFMI cutoff values for predicting low muscle mass are <18 kg/m^2^ in men and <15 kg/m^2^ in women [12]. Thus, in the current analysis, reduced FFMI was defined as FFMI <18 kg/m^2^ in men and <15 kg/m^2^ in women. Based on these thresholds, 1674 men (40.1%) and 2160 women (47.3%) were classified as having reduced FFMI. All measurements were obtained during the same clinical visit at which demographic characteristics and laboratory variables were collected.

In the present study, FFMI was used as the primary indicator of muscle mass because it provides a standardized measure of FFM adjusted for body size and has been proposed as a surrogate marker for low muscle mass screening. As mentioned above, low FFMI was defined as FFMI <18 kg/m^2^ in men and <15 kg/m^2^ in women. Participants were subsequently classified into low-FFMI and non-low-FFMI groups based on these sex-specific thresholds.

### 2.3. Predictor Variable Preprocessing and Feature Selection

Prior to feature selection, a preprocessing procedure was performed to reduce redundancy and multicollinearity among candidate predictors. For variables derived from other clinical measurements, only the composite indices were retained while their component variables were excluded. Specifically, body weight and height were excluded because they were incorporated into BMI or FFMI. Also, to maintain a parsimonious and consistent predictor set across the male and female models, eGFR was retained in the primary model, while an alternative model including BMI, age, and creatinine was evaluated in a sensitivity analysis (Table S3). eGFR was calculated from measured serum creatinine using the three-variable Japanese equation: $eGFR\;=\;194\;\times \;Cr^{-1.094}\times \;age^{-0.287}\times \;0.739$ (if female). To further reduce multicollinearity, correlation analyses were conducted separately for men and women using Pearson’s correlation coefficients (Figure 1B). All candidate predictors are listed in Table 1. Pairs of variables with an absolute Pearson correlation coefficient of |r| ≥ 0.80 were considered redundant, with the sex-specific correlated pairs shown in Figure 1B. One variable from each pair was retained based on clinical interpretability and practical applicability. These preprocessing decisions were made using the full dataset before the repeated resampling procedures. Variables selected for retention were preferentially chosen according to their clinical relevance and ease of implementation in routine clinical practice.

**Figure 2:**
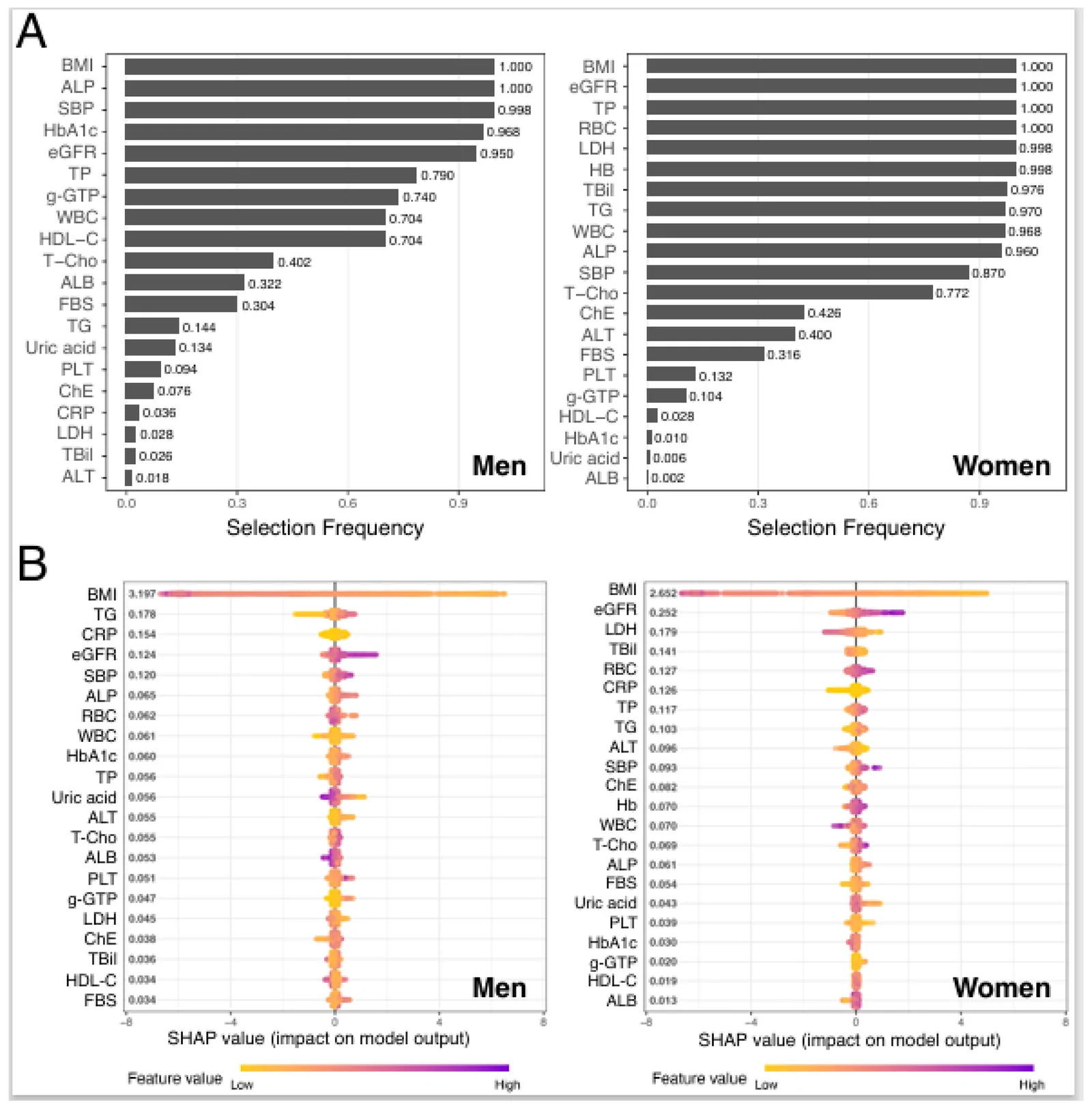
**Feature selection using LASSO and SHAP analyses in men and women:** (**A**) Selection frequency of candidate variables based on repeated LASSO logistic regression (500 iterations). Bars represent the proportion of iterations in which each variable was selected, with higher values indicating greater stability and importance. Variables are ordered by selection frequency. (**B**) SHAP summary plots from XGBoost classification models used to distinguish individuals with low FFMI, defined as FFMI below the sex-specific cutoff (18 kg/m^2^ for men and 15 kg/m^2^ for women [12]). Each point represents an individual; the x-axis shows the SHAP value (feature contribution to the model output), and color indicates the feature value (low to high). Features are ranked by overall importance. The numbers shown next to each feature represent the mean absolute SHAP values, indicating the overall contribution of each variable to the model. Alb, albumin; ALP, alkaline phosphatase; ALT, alanine aminotransferase; AST, aspartate aminotransferase; BIA, bioelectrical impedance analysis; BMI, body mass index; ChE, cholinesterase; CRP, C-reactive protein; DBP, diastolic blood pressure; eGFR, estimated glomerular filtration rate; FBS, fasting blood glucose; FFMI, fat-free mass index; Hb, hemoglobin; HbA1c, hemoglobin A1c; HDL-C, high-density lipoprotein cholesterol; Ht, hematocrit; LASSO, least absolute shrinkage and selection operator; LDH, lactate dehydrogenase; LDL-C, low-density lipoprotein cholesterol; PLT, platelet count; RBC, red blood cell count; ROC, receiver operating characteristic; SBP, systolic blood pressure; SHAP, SHapley Additive exPlanations; TBil, total bilirubin; T-Cho, total cholesterol; TG, triglycerides; TP, total protein; WBC, white blood cell count; WC, waist circumference; XGBoost, extreme gradient boosting; γ-GTP, gamma-glutamyl transferase.

Feature selection was subsequently performed independently using both LASSO regression and SHAP analysis (Figure 2). For the LASSO analysis, the outcome variable was defined as low FFMI according to the sex-specific cutoffs. The dataset was randomly divided into training (80%) and test (20%) sets, and LASSO logistic regression models were fitted using the “cv.glmnet” function in R (version 4.3.0). This procedure was repeated 500 times, and the selection frequency of each variable was calculated as the proportion of iterations in which the corresponding coefficient remained non-zero. Variables with selection frequencies greater than 0.9 were considered highly stable predictors (Figure 2A).

To evaluate feature importance from a machine-learning perspective, the data for each sex were divided into training (80%) and held-out test (20%) sets. XGBoost hyperparameters were tuned within the training set using grid search with five-fold cross-validation. Random seeds of 42 and 43 were used for the analyses in men and women, respectively. The grid included maximum tree depths of 2, 4, and 6; learning rates of 0.03 and 0.10; and minimum child weights of 1 and 5, while subsample and column-subsample ratios were fixed at 0.8. A binary logistic objective and AUC evaluation metric were used without class weighting. The number of boosting rounds was selected using early stopping after 20 rounds without improvement, with a maximum of 500 rounds. SHAP values were calculated in the held-out test observations, and variables were ranked according to their mean absolute SHAP values (Figure 2B). Final predictors were defined as variables with a LASSO selection frequency greater than 0.90 that also ranked among the top five mean absolute SHAP features in both sexes.

### 2.4. Development and Validation of the Logistic Regression Model

Based on the feature-selection analyses, BMI and eGFR were selected as the final predictors. Sex-specific logistic regression models were constructed using BMI and eGFR as explanatory variables and low FFMI status as the outcome. The models were fitted in R (version 4.3.0) using the glm function.

**Figure 3:**
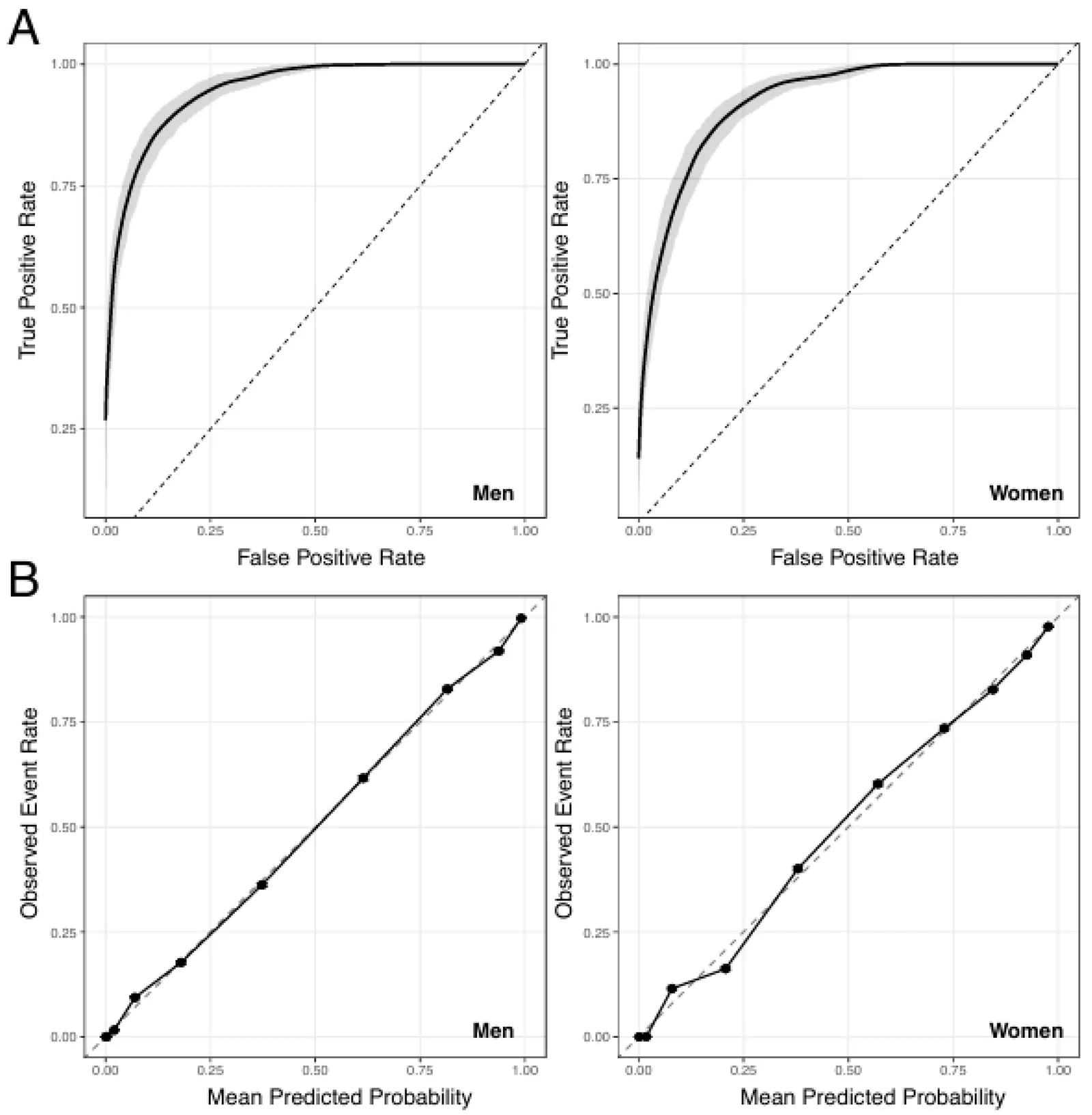
**Performance and calibration of the logistic regression model in men and women:** (**A**) Receiver operating characteristic (ROC) curves of the logistic regression model for predicting low FFMI in men and women. The solid line represents the mean ROC curve obtained from 500 repeated random train-test splits, and the shaded area indicates the 2.5th–97.5th percentile empirical interval across the 500 repeated splits. The dashed diagonal line represents no discrimination. (**B**) Calibration plots of the logistic regression model comparing predicted probabilities with observed event rates. Held-out predicted probabilities from the repeated test sets were pooled and divided into 10 equally sized groups according to predicted probability. Each point represents the mean predicted probability and the corresponding observed prevalence of low FFMI within a group.

Model performance was evaluated using 500 repeated stratified 8:2 train–test splits. In each repetition, the model was fitted using the training set and evaluated using the corresponding held-out test set. Discrimination was assessed using the area under the receiver operating characteristic curve (AUC) (Figure 3A). The optimal Youden cut-off was determined exclusively from the training set and applied to the corresponding test set to estimate sensitivity and specificity. AUC, sensitivity, specificity, Brier score, calibration intercept, and calibration slope were summarized using the mean and 2.5th–97.5th percentile empirical interval across the 500 repetitions, whereas the Youden cut-off was summarized using the median and corresponding empirical interval. Because participants could appear in multiple training and test sets, these percentile ranges were interpreted as descriptive empirical intervals rather than conventional confidence intervals based on independent observations.

Calibration was evaluated using held-out test-set predictions. For graphical assessment, pooled held-out predictions were divided into 10 equally sized groups according to predicted probability, and the mean predicted probability was compared with the observed prevalence of low FFMI within each group (Figure 3B).

After validation, the final sex-specific logistic regression models were reconstructed using the entire dataset solely to obtain the final model coefficients and prediction equations. Model performance and risk-stratification analyses were based on held-out predictions.

**Figure 4:**
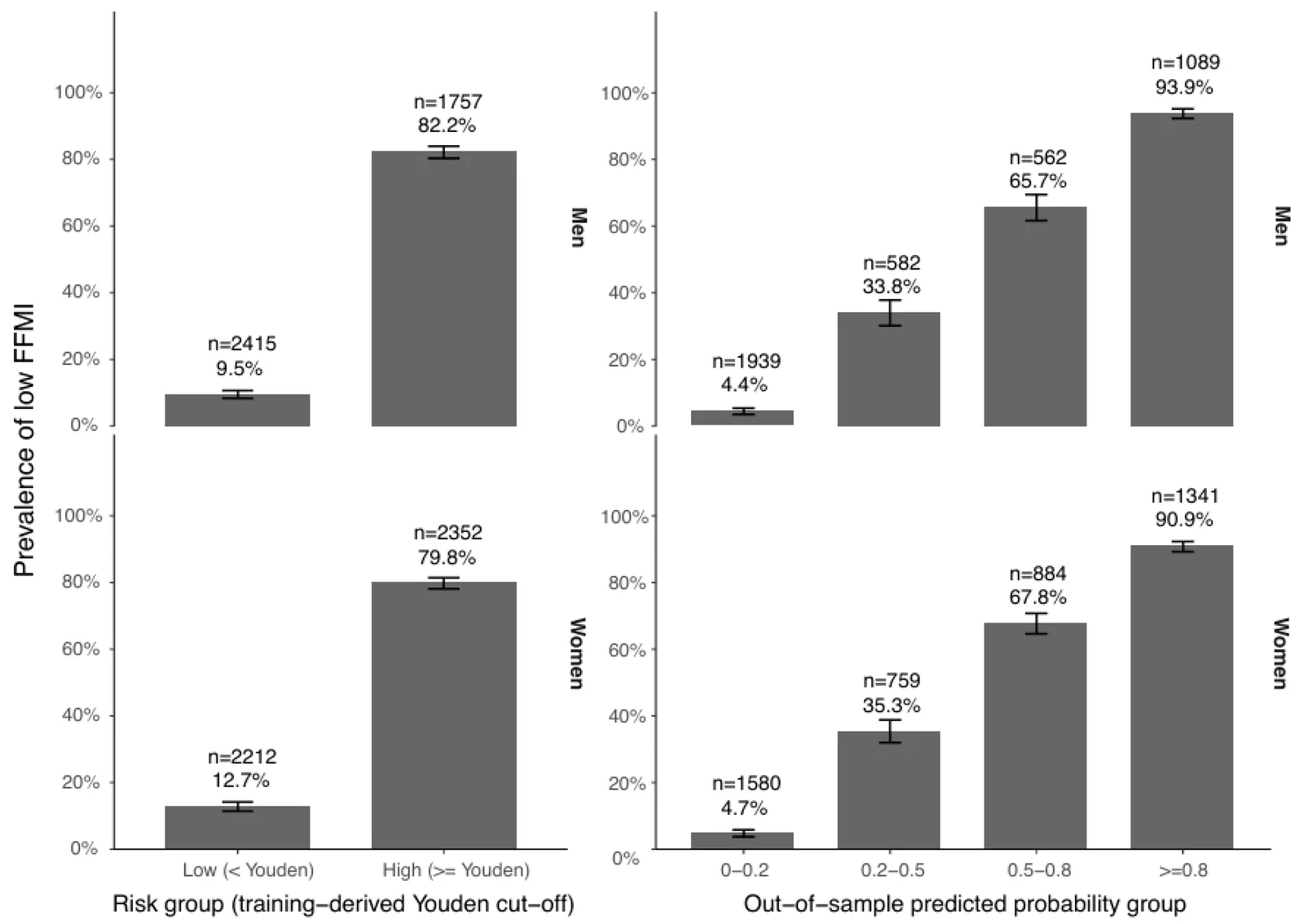
**Risk stratification of the logistic regression model in men and women:** For the Youden-based groups (**left**), each held-out prediction was classified using the cut-off derived from the corresponding training set, and the participant-level group was assigned according to the majority of held-out classifications. For the fixed probability categories (**right**), held-out predicted probabilities were averaged for each participant and categorized as <0.2, 0.2–<0.5, 0.5–<0.8, or ≥0.8. Bars represent the observed prevalence of low FFMI, error bars indicate Wilson 95% confidence intervals, and labels show the sample size and prevalence. The fixed categories were exploratory and did not represent validated clinical decision thresholds. FFMI; fat-free mass index.

For participant-level risk stratification, each held-out prediction was classified using the corresponding training-derived Youden cut-off, and the participant-level Youden group was assigned according to the majority of held-out classifications. For the fixed probability categories, predicted probabilities were averaged for each participant across the repetitions in which the participant was included in the held-out test set. The resulting participant-level out-of-sample probabilities were categorized as <0.2, 0.2–<0.5, 0.5–<0.8, and ≥0.8 (Figure 4). These categories were used for exploratory descriptive risk stratification and were not intended to represent validated clinical decision thresholds.

As a sensitivity analysis, the finalized BMI–eGFR model was additionally evaluated in all participants with available FFMI, BMI, and eGFR, irrespective of missingness in other candidate variables (Table S4). As a separate internal descriptive analysis, continuous FFMI was estimated separately in men and women using LASSO linear regression with BMI and eGFR as predictors, and Pearson’s correlation coefficient was calculated between estimated and BIA-measured FFMI (Figure S1). This analysis was not used to validate the logistic model.

### 2.5. Assessment of the Incremental Value of eGFR Beyond BMI

Because BMI and FFMI share height squared as the denominator and body weight includes fat-free mass, BMI is mathematically coupled with the outcome. To quantify the predictive value of eGFR beyond BMI, a BMI-only logistic regression model was compared with the BMI–eGFR model separately in men and women.

Both models were evaluated using 500 repeated stratified 8:2 train–test splits. Within each repetition, identical training and test samples were used for the two models. Test-set performance was assessed using AUC, Brier score, calibration intercept, and calibration slope. Paired differences in AUC and Brier score were calculated within each repetition and summarized using their means and 2.5th–97.5th percentile empirical intervals.

Because the BMI-only model was nested within the BMI–eGFR model, improvement in model fit after adding eGFR was additionally assessed using a likelihood ratio test in the complete dataset.

Clinical utility was evaluated using internally validated decision-curve analysis based exclusively on held-out test-set predictions. The intended clinical decision was whether to recommend confirmatory BIA measurement. Lower threshold probabilities prioritize sensitivity but may increase unnecessary BIA measurements, whereas higher thresholds reduce unnecessary measurements at the cost of missing some individuals with low FFMI. Decision curves were evaluated over the threshold probability range of 0.05–0.50. At each threshold, the difference in net benefit between the BMI–eGFR and BMI-only models was summarized using the mean and 2.5th–97.5th percentile empirical interval across the 500 repetitions (Figure S2).

### 2.6. Statistical Analysis

Continuous variables are presented as mean (SD), and categorical variables are presented as frequencies and percentages. Continuous predictors were entered on their original scales without additional transformation unless otherwise specified. No additional outlier exclusion or winsorization was performed. No class weighting was applied, and stratified sampling was used to preserve the outcome distribution in repeated train–test splits. Model discrimination, calibration, empirical intervals, and decision-curve analysis were performed as described in Section 2.4 and Section 2.5. All statistical analyses and model development procedures were performed using R software (version 4.3.0) with the glmnet, xgboost, SHAPforxgboost, and pROC packages. A two-sided *p* value <0.05 was considered statistically significant.

## 3. Results

### 3.1. Selection and Handling of Predictor Variables

To minimize the risk of overfitting in subsequent predictive analyses, a preprocessing and variable selection procedure was performed prior to model development. First, for clinically derived indices calculated from combinations of other variables, only the final composite index was retained while the original component variables were removed to reduce redundancy and multicollinearity. For example, BMI was retained while body weight and height were excluded. Similarly, eGFR was retained in the primary model, while age and Cr were evaluated separately in sensitivity analysis.

In addition, correlation analyses among predictor variables were separately performed for men and women. When the Pearson correlation coefficient between two variables was |r| ≥ 0.80, the variables were considered highly collinear, and only one representative variable was retained based on clinical interpretability and practical relevance (Figure 1B).

### 3.2. Variable Selection and Identification of Key Predictors

To develop a clinically practical prediction model while maintaining predictive performance, we aimed to minimize the number of clinical variables used in the model. Selecting a limited number of key predictors can improve model parsimony and reduce overfitting, although removing informative predictors may also reduce predictive accuracy.

To this end, we performed both LASSO regression, which assumes linear relationships, and SHAP analysis based on an XGBoost model, which can capture nonlinear relationships and interactions among variables, to identify predictors consistently considered important across both approaches. First, to evaluate the stability of variable selection, LASSO regression models were repeatedly constructed using randomly sampled subsets comprising 80% of the entire dataset, and this procedure was repeated 500 times. The selection frequency of each variable was then calculated. In men, 5 variables (BMI, alkaline phosphatase, systolic blood pressure, HbA1c, and eGFR) showed selection frequencies greater than 0.9. In women, 10 variables (BMI, eGFR, total protein, red blood cell, LDH, hemoglobin, total bilirubin, triglyceride, white blood cell, and alkaline phosphatase) demonstrated selection frequencies greater than 0.9 (Figure 2A).

Next, feature importance was evaluated using SHAP analysis based on the XGBoost model. In men, the top five variables were BMI, triglyceride, CRP, eGFR, and systolic blood pressure, whereas in women, the most important variables were BMI, eGFR, LDH, total bilirubin, and triglyceride (Figure 2B). Notably, BMI showed the highest importance in both sexes, and eGFR was also consistently identified as an important predictor in both men and women (Figure 2A,B).

Final predictors were required to have a LASSO selection frequency greater than 0.90 and to rank among the top five features by mean absolute SHAP value in both sexes. Only BMI and eGFR met both criteria and were therefore selected as the final predictors. Clinically, BMI is a representative body composition-related index derived from body weight and height, whereas eGFR reflects renal function and is calculated using age and serum Cr levels, supporting the clinical interpretability and applicability of these predictors.

### 3.3. Development and Validation of the BMI–eGFR Risk Model and Comparison with BMI Alone

Next, sex-specific logistic regression models were constructed using BMI and eGFR selected in Figure 2 to predict decreased muscle mass based on FFMI criteria [12]. Model validity was assessed through discrimination and calibration. To evaluate model performance, the dataset was randomly divided into training and test sets at an 8:2 ratio, and the procedure of training the model on 80% of the data and evaluating performance on the remaining 20% was repeated 500 times (Figure 3A). Predictive performance was assessed using ROC-AUC analysis. Across 500 repeated train–test splits, the mean AUC was 0.946 in men and 0.918 in women. Using Youden cut-offs determined exclusively from the training sets, the mean sensitivity and specificity in the corresponding test sets were 0.861 and 0.875 in men and 0.869 and 0.805 in women, respectively. Brier scores and calibration measures also indicated good overall model performance (Table S5).

Finally, logistic regression models were reconstructed using the entire dataset. The resulting equations were as follows: for men, η=29.98−1.36×BMI+0.011×eGFR, and for women, η=18.20−0.98×BMI+0.031×eGFR, where logistic regression model is defined by

$$p=\frac{1}{1+\exp\left(-\eta \right)}.\;$$

Here, η denotes the sex-specific linear predictor and p=1/[1+exp(−η)] represents the predicted probability of low FFMI. BMI is expressed in kg/m^2^ and eGFR in mL/min/1.73 m^2^. Coefficients were rounded to two decimal places, except for the smaller eGFR coefficients, which were presented to three decimal places. Complete regression results, including coefficients, standard errors, odds ratios, 95% confidence intervals, and *p* values, are presented in Table S6.

Calibration was evaluated using held-out test-set predictions. The Brier score, calibration intercept, and calibration slope indicated good overall calibration in both sexes, with their distributions across the 500 repetitions presented in Table S5. The decile-based calibration plots were generally consistent with these numerical findings (Figure 3B). To assess the incremental predictive value of eGFR beyond BMI, the BMI-only and BMI–eGFR models were directly compared using identical training and test samples across the 500 repeated train–test splits. In men, the mean test-set AUC was 0.945 for the BMI-only model and 0.946 for the BMI–eGFR model. The mean change in AUC was 0.0004 (2.5th–97.5th percentile empirical interval, −0.0009 to 0.0011). The corresponding Brier scores were 0.0922 and 0.0918, respectively. Calibration intercepts were 0.0076 and 0.0079, and calibration slopes were both approximately 1.01, indicating minimal change in calibration after adding eGFR. In women, the mean AUC increased from 0.914 for the BMI-only model to 0.918 for the BMI–eGFR model. The mean change in AUC was 0.0046 (2.5th–97.5th percentile empirical interval, 0.0003–0.0087), and the Brier score decreased from 0.118 to 0.115. Calibration intercepts and slopes remained similar between the two models. Addition of eGFR significantly improved model fit in the likelihood ratio tests in both men $\chi^{2}\left(1\right)=7.92$, P=0.0049 and women $\chi^{2}\left(1\right)=89.56$, P<0.001. However, the incremental improvements in test-set discrimination, overall prediction error, and calibration were small, particularly in men. For reference, the eGFR-only model showed substantially lower discrimination in both sexes.

In a separate internal descriptive analysis, continuous FFMI values estimated from BMI and eGFR using sex-specific LASSO regression showed a close empirical relationship with BIA-measured FFMI in both sexes (Figure S1). Because BMI and FFMI are mathematically coupled, this analysis was not interpreted as evidence of independent predictive validity but as a descriptive illustration of the relationship between routinely obtainable measurements and FFMI.

Furthermore, the expanded model-available cohort included 17,673 participants (7647 men and 10,026 women). The mean AUCs were 0.951 in men and 0.922 in women, compared with 0.946 and 0.918, respectively, in the complete-case cohort; Brier scores and calibration metrics were also comparable between the two cohorts (Table S4).

The alternative BMI–age–creatinine model showed AUC, Brier score, and calibration comparable to those of the BMI–eGFR model in both sexes (Table S3).

### 3.4. Prevalence of Low FFMI According to Predicted Risk

We evaluated risk stratification using participant-level out-of-sample predictions (Figure 4). The median training-derived Youden cut-offs were 0.436 in men (2.5th–97.5th percentile empirical interval, 0.377–0.473) and 0.450 in women (0.408–0.487). The prevalence of low FFMI in the low- and high-risk groups was 9.5% and 82.2% in men and 12.7% and 79.8% in women, respectively. Across the four descriptive probability categories, the prevalence increased progressively from 4.4%, 33.8%, 65.7%, to 93.9% in men and from 4.7%, 35.3%, 67.8%, to 90.9% in women. These categories illustrated the gradient of predicted risk but were not interpreted as validated clinical decision thresholds.

### 3.5. Decision-Curve Analysis of the BMI-Only and BMI–eGFR Models

**Figure 5:**
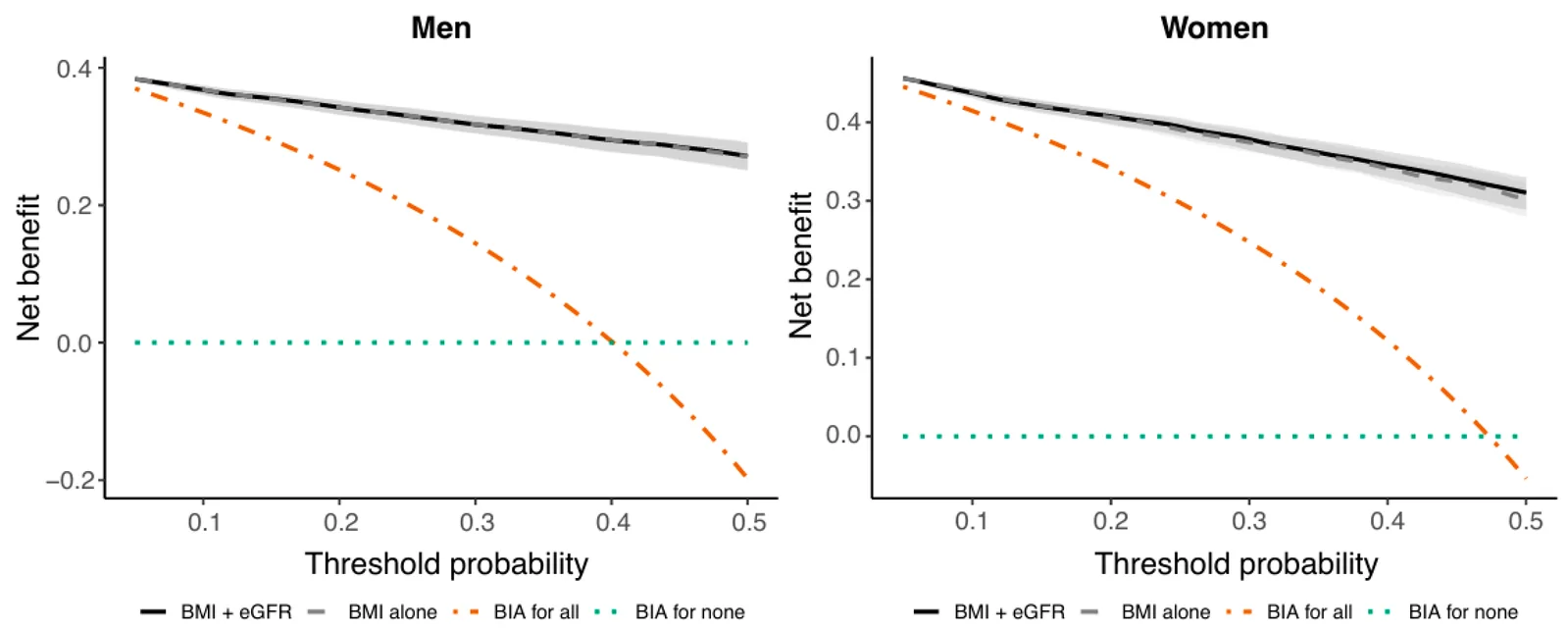
**Internally validated decision-curve analysis comparing the BMI-only and BMI–eGFR models:** The clinical decision was whether to perform confirmatory BIA measurement. The curves represent the mean net benefit calculated from held-out test-set predictions across 500 repeated stratified 8:2 train–test splits. Shaded areas represent the 2.5th–97.5th percentile empirical intervals. The reference strategies indicate BIA measurement for all participants and BIA measurement for no participants. The complete threshold probability range of 0.05–0.50 is shown.

Finally, decision curve analysis (DCA) was performed to evaluate the potential clinical utility of the BMI-eGFR–based risk model (Figure 5). In the internally validated decision-curve analysis, both the BMI-only and BMI–eGFR models showed higher mean net benefit than the strategies of performing BIA for all participants or for no participants over most of the evaluated threshold range of 0.05–0.50 (Figure 5).

In men, the decision curves of the BMI-only and BMI–eGFR models almost completely overlapped, and no consistent incremental net benefit was observed after adding eGFR. In women, the BMI–eGFR model showed a small increase in mean net benefit at threshold probabilities of approximately 0.20 or higher; however, the 2.5th–97.5th percentile empirical intervals for the incremental net benefit included zero (Figure S2).

## 4. Discussion

Sarcopenia can be a factor contributing to poor prognosis, and in Japan, where an aging society is already a reality, it is a significant predictor of poor prognosis [1,13,14]. AWGS2019 recommended assessing grip strength, muscle mass, and physical function to determine the severity of sarcopenia [6]. On the other hand, AWGS2025 diagnoses sarcopenia based solely on measurements of grip strength and muscle mass, highlighting the growing importance of assessing muscle mass [5]. However, not all facilities are able to measure muscle mass for the purpose of assessing sarcopenia. AWGS2019 and AWGS2025 employ a two-track algorithm, dividing the settings into community settings and hospital or research settings [5,6]. At facilities unable to measure muscle mass, cases with grip strength or physical function below the reference values are considered suspected cases of sarcopenia, and intervention is recommended for such cases. Using data from a large number of screening participants, we developed a formula based on two factors—BMI and eGFR—that can be easily measured in routine clinical practice, and found it to be a useful screening tool for low FFMI in a cohort spanning a broad adult age range. One of the key features of this formula is that it can be applied to younger individuals (mean age: 55.08 years for men and 53.33 years for women in this study). It is well known that skeletal muscle mass in humans begins to decline at a rate of about 1% per year starting around the age of 50 [15]. The AWGS2025 guidelines also include new diagnostic criteria for sarcopenia in subjects under the age of 65 [5].

The association between lower BMI and lower FFMI is biologically plausible, as BMI well reflects body composition and nutritional status. On the other hand, renal dysfunction may be associated with chronic systemic inflammation and metabolic stress, which are known to activate catabolic signaling pathways including NF-κB signaling and JAK-STAT signaling, ultimately promoting muscle protein degradation through the ubiquitin-proteasome pathway [16]. These mechanistic connections support the clinical utility of BMI and eGFR as surrogate markers for muscle mass assessment. Furthermore, chronic low-grade inflammation, which is common in patients with chronic kidney disease, may contribute to muscle atrophy through dysregulated cytokine signaling. The IL-6 pathway exemplifies this duality: while acute exercise-induced IL-6 elevation promotes metabolic health and muscle adaptation, persistent IL-6 elevation in chronic disease status drives muscle catabolism via JAK-STAT signaling-mediated protein degradation [2,17].

In our results, in both men and women, the predicted probabilities by our formula showed good agreement with the observed event rates, and the calibration curves were generally close to the reference line. Repeated held-out validation yielded mean AUCs of 0.946 in men and 0.918 in women, with mean sensitivities and specificities of 0.861/0.875 and 0.869/0.805, respectively. Participant-level out-of-sample predictions also demonstrated clear stratification of the observed prevalence of low FFMI. The internally validated DCA suggested potential utility for prioritizing confirmatory BIA measurement; however, external or prospective validation is required before clinical benefit can be established.

The AWGS2025 recommends the use of the finger-circle test as a case-finding method [5]. The finger-circle test is a simple self-assessment method for evaluating the risk of sarcopenia by wrapping a loop formed by the thumb and index finger of both hands around the calf [18]. A three-tier assessment is conducted based on whether the ring fits loosely around the calf (Bigger), fits snugly (Just-fit), or leaves a gap (Smaller); a gap is considered a strong indicator of sarcopenia. Hiraoka et al. investigated the correlation between the psoas muscle index (PMI) on CT and the finger-circle test in patients with liver disease [19]. In their ROC analyses, the sensitivity/specificity of a finger-circle test for reduced PMI was 0.444–0.740/0.583–0.776, and the AUCs of a finger-circle test for reduced PMI was 0.612–0.698 [19]. In a study of elderly Thai people, the sensitivity/specificity of the finger-circle test for sarcopenia in men were 0.857/0.712 (AUC = 0.785), and for women, the sensitivity/specificity of the test were 0.875/0.808 (AUC = 0.842) [20]. In a study in Singapore of community dwelling elderly people, the finger-circle test had diagnostic performance in determining decreased muscle mass (AUC = 0.591, sensitivity/specificity = 0.571/0.584) and sarcopenia diagnosis (AUC = 0.581, sensitivity/specificity = 0.556/0.744) [21]. Although it may be difficult to directly compare our findings with those of previous studies, the results of our ROC analysis suggest that our formula for identifying low FFMI is helpful in daily clinical practice. On the other hand, the SARC-F is a self-administered screening tool that allows for the simple assessment of the risk of sarcopenia in older adults by answering just five questions: strength, assistance in walking, rising from a chair, climbing stairs, and falls [18]. A study of elderly Japanese individuals reported that the SARC-F had a sensitivity of 0.417 and a specificity of 0.685 for sarcopenia [22]; given its low sensitivity, the SARC-F appears to be problematic as a screening method [23]. In contrast, as mentioned above, our formula demonstrates good sensitivity for identifying low FFMI.

A Chinese study reported that lower BMI was significantly associated with higher odds of decreased muscle mass (adjusted odds ratio = 0.508), while higher BMI exhibited a protective effect [24]. In this study, the percentage of cases with a BMI of 25 kg/m^2^ or higher and decreased muscle mass was 4.3% among men and 2.0% among women. It should be noted that this formula may not be applicable to cases such as sarcopenic obesity, where there is a significant discrepancy between fat mass and muscle mass [25]. It is also important to note that conditions such as ascites can cause an apparent increase in BMI. A previous meta-analysis reported that sarcopenia was significantly associated with urinary albumin or protein level and decreased eGFR [26]. In cases where renal function is changing rapidly, or in severely ill patients whose general condition is fluctuating significantly, the accuracy of eGFR values may be compromised, and caution is required when using our formula for muscle mass assessment [27]. Severe dehydration status can also affect BMI and eGFR. Because serum creatinine is influenced by muscle mass, eGFR may also partly reflect the target condition and should not be regarded as fully independent of FFMI. Although eGFR does not substitute for the independent biological effect of age, it was retained to preserve a parsimonious and common predictor set across both sexes because the alternative BMI–age–creatinine model did not materially improve predictive performance.

As mentioned earlier, AWGS2019 and AWGS2025 employ a two-track algorithm, dividing the settings into community settings and hospital or research settings. By incorporating our formula into cases where muscle mass cannot be measured, we can identify individuals who may benefit from confirmatory muscle-mass assessment more efficiently. Furthermore, the values obtained from our formula make it possible to estimate the risk of decreased muscle mass. It is also expected to reduce healthcare costs in the future [28].

The current study was conducted on Japanese subjects and therefore has limitations in that the findings may not apply to other ethnic groups, and our formula may also not apply to cases involving severe underlying diseases. In addition, the current study provides only internal validation using repeated random splits from the same single-center cohort, and the external validation was not conducted. A significant number of participants were excluded from the analysis, and muscle strength was not measured. Finally, FFMI includes all fat-free tissue and is not completely equivalent to skeletal muscle mass. All of these limitations may introduce bias. Moving forward, we plan to investigate whether our formula is useful for assessing muscle mass in patients with underlying medical conditions such as malignancies, metabolic diseases, cardiovascular disease, etc. Also, predictor selection was informed by analyses of the full cohort because our objective was to identify a single parsimonious predictor set applicable to both sex-specific models, rather than to develop an automated feature-selection pipeline. Therefore, although the finalized BMI–eGFR model was evaluated using repeated train–test splits, some optimism arising from data-informed predictor selection cannot be excluded. Although the sensitivity analysis in the expanded cohort with available FFMI, BMI, and eGFR yielded comparable results (Table S4), residual selection bias arising from the complete-case analysis cannot be excluded.

## 5. Conclusions

In conclusion, our formula is expected to prove useful for estimating low fat-free mass, particularly in situations where muscle mass cannot be measured.

## Figures and Tables

**Table 1:** Baseline characteristics of the study cohort.

Characteristic	Male (n = 4172)	Female (n = 4564)	Total (n = 8736)
**Age, years**	55.08 (12.13)	53.33 (11.38)	54.17 (11.77)
**Height, cm**	170.53 (6.24)	157.82 (5.77)	163.89 (8.73)
**Weight, kg**	69.03 (11.17)	54.04 (9.36)	61.20 (12.70)
**Body mass index, kg/m^2^**	23.70 (3.39)	21.69 (3.54)	22.65 (3.62)
**Waist circumference, cm**	85.18 (9.32)	78.11 (9.50)	81.48 (10.06)
**White blood cell count, ×10^3^/μL**	5.54 (1.68)	5.09 (1.35)	5.30 (1.54)
**Red blood cell count, ×10^6^/μL**	483.90 (42.02)	440.09 (35.43)	461.01 (44.47)
**Platelet count, ×10^3^/μL**	24.34 (5.44)	25.71 (6.27)	25.06 (5.93)
**Hemoglobin, g/dL**	14.86 (1.08)	13.10 (1.13)	13.94 (1.41)
**Hematocrit, %**	44.47 (3.06)	40.06 (3.04)	42.17 (3.76)
**Total cholesterol, mg/dL**	206.04 (32.54)	216.60 (35.25)	211.56 (34.39)
**HDL cholesterol, mg/dL**	63.37 (16.81)	78.35 (17.87)	71.20 (18.91)
**LDL cholesterol, mg/dL**	120.25 (29.38)	121.89 (30.81)	121.11 (30.15)
**Triglycerides, mg/dL**	112.06 (61.48)	81.81 (42.75)	96.26 (54.66)
**Fasting blood glucose, mg/dL**	96.26 (16.86)	89.73 (12.18)	92.85 (14.96)
**Hemoglobin A1c, %**	5.77 (0.59)	5.67 (0.42)	5.72 (0.51)
**Systolic blood pressure, mmHg**	122.37 (16.12)	116.82 (17.70)	119.47 (17.19)
**Diastolic blood pressure, mmHg**	79.14 (11.77)	72.34 (12.15)	75.59 (12.44)
**Total protein, g/dL**	7.18 (0.37)	7.19 (0.37)	7.19 (0.37)
**Serum a** **lbumin, g/dL**	4.38 (0.26)	4.30 (0.24)	4.34 (0.25)
**Total bilirubin, mg/dL**	0.91 (0.36)	0.78 (0.30)	0.84 (0.34)
**Aspartate aminotransferase, IU/L**	24.10 (10.50)	21.33 (7.13)	22.65 (9.01)
**Alanine aminotransferase, IU/L**	25.69 (17.95)	17.81 (10.91)	21.57 (15.22)
**Gamma-glutamyl transferase, IU/L**	43.65 (48.55)	24.37 (26.17)	33.58 (39.70)
**Uric acid, mg/dL**	6.13 (1.21)	4.62 (1.02)	5.34 (1.34)
**Alkaline phosphatase, IU/L**	68.10 (17.97)	64.85 (20.13)	66.40 (19.20)
**Cholinesterase, IU/L**	345.84 (68.68)	320.63 (74.62)	332.67 (72.94)
**Lactate dehydrogenase, IU/L**	169.99 (29.30)	171.23 (30.85)	170.64 (30.12)
**C-reactive protein, mg/dL**	0.11 (0.33)	0.08 (0.27)	0.10 (0.30)
**Estimated glomerular filtration rate, mL/min/1.73 m^2^**	70.07 (13.21)	73.48 (13.63)	71.85 (13.54)
**Low Fat-Free Mass Index, n (%)**	1674 (40.1)	2160 (47.3)	3834 (43.9)

Data are presented as mean (standard deviation) or n (%).

## Data Availability

The original contributions presented in this study are included in the article/Supplementary Material. Further inquiries can be directed to the corresponding author(s).

## Supplementary Materials

The following supporting information can be downloaded at https://www.mdpi.com/article/10.3390/jcm15166431/s1: Figure S1: Correlation between measured and LASSO-estimated FFMI: Measured fat-free mass index (FFMI) was plotted against FFMI estimated by LASSO regression using BMI and eGFR in men (left) and women (right). Each dot represents one participant, and the red dashed line indicates the identity line. The correlations between measured and estimated FFMI were 0.91 in men and 0.87 in women, respectively; Figure S2: Incremental net benefit of adding eGFR to the BMI-only model. Incremental net benefit was calculated as the net benefit of the BMI–eGFR model minus that of the BMI-only model at each threshold probability using held-out test-set predictions from 500 repeated train–test splits. The solid line represents the mean incremental net benefit, and the shaded area represents the 2.5th–97.5th percentile empirical interval across repetitions. The dashed horizontal line at zero indicates no difference in net benefit between the two models. BMI, body mass index; eGFR, estimated glomerular filtration rate; Table S1: Variable-specific missingness in the initially identified cohort; Table S2: Comparison of participants included in and excluded from the complete-case cohort; Table S3: Sensitivity analysis comparing the BMI–eGFR model with the BMI–age–creatinine model; Table S4: Sensitivity analysis in the expanded model-available cohort; Table S5: Internally validated performance of the BMI–eGFR model; Table S6: Complete sex-specific logistic regression results for the final BMI–eGFR model.

## Abbreviations

AWGS; Asian Working Group for Sarcopenia, FFMI; fat-free mass index, BMI; body mass index, eGFR; estimated glomerular filtration rate, FFM; fat-free mass (FFM), BIA; bioelectrical impedance analysis, Cr; creatinine, AUC; area under the receiver operating characteristic curve, SD; standard deviation, DCA; decision curve analysis, PMI; psoas muscle index.
