# Supplementary material for: Development of a Simple Formula for Identifying Reduced Fat-Free Mass in Health Screening Participants
Source: J Clin Med. 2026 Aug 20;15(16):6431. doi: 10.3390/jcm15166431 (PMC13514340; doi:10.3390/jcm15166431)
Supplement: Supplementary file 1 [file jcm-15-06431-s001.zip › jcm-4462174-supplementary.pdf]

Supplementary Information

Development of a Simple Formula for Identifying Reduced Fat-Free Mass in Health Screening Participants

Hiroki Nishikawa [1,†], Hyeongki Park [2,3,†], Raiki Yoshimura [3], Shingo Iwami [3], Akira Asai [1], Soo Ki Kim [4], Akira Fukuda [5] and Sachiyo Yoshio [6,*]

[1] The Second Department of Internal Medicine, Osaka Medical and Pharmaceutical University, Takatsuki 569-8686, Osaka, Japan; hiroki.nishikawa@ompu.ac.jp (H.N.); akira.asai@ompu.ac.jp (A.A.)
[2] School of Biomedical Convergence Engineering, Pusan National University, Yangsan 50612, Republic of Korea; parkhyeongki0226@gmail.com
[3] Interdisciplinary Biology Laboratory (iBLab), Division of Natural Science, Graduate School of Science, Nagoya University, Nagoya 464-8601, Aichi, Japan; yoshimura.raiki21@gmail.com (R.Y.); iwamishingo@gmail.com (S.I.)
[4] Department of Gastroenterology, Kobe Asahi Hospital, Kobe 653-8501, Hyogo, Japan; kinggold@kobe-asahi-hp.com
[5] Health Science Clinic, Osaka Medical and Pharmaceutical University, Takatsuki 569-8686, Osaka, Japan; akira.fukuda@ompu.ac.jp
[6] Department of Human Immunology and Translational Research, National Institute of Global Health and Medicine, Japan Institute for Health Security, Shinjukuku 162-0052, Tokyo, Japan
* Correspondence: yoshio.s@jihs.go.jp
† These authors contributed equally to this study.

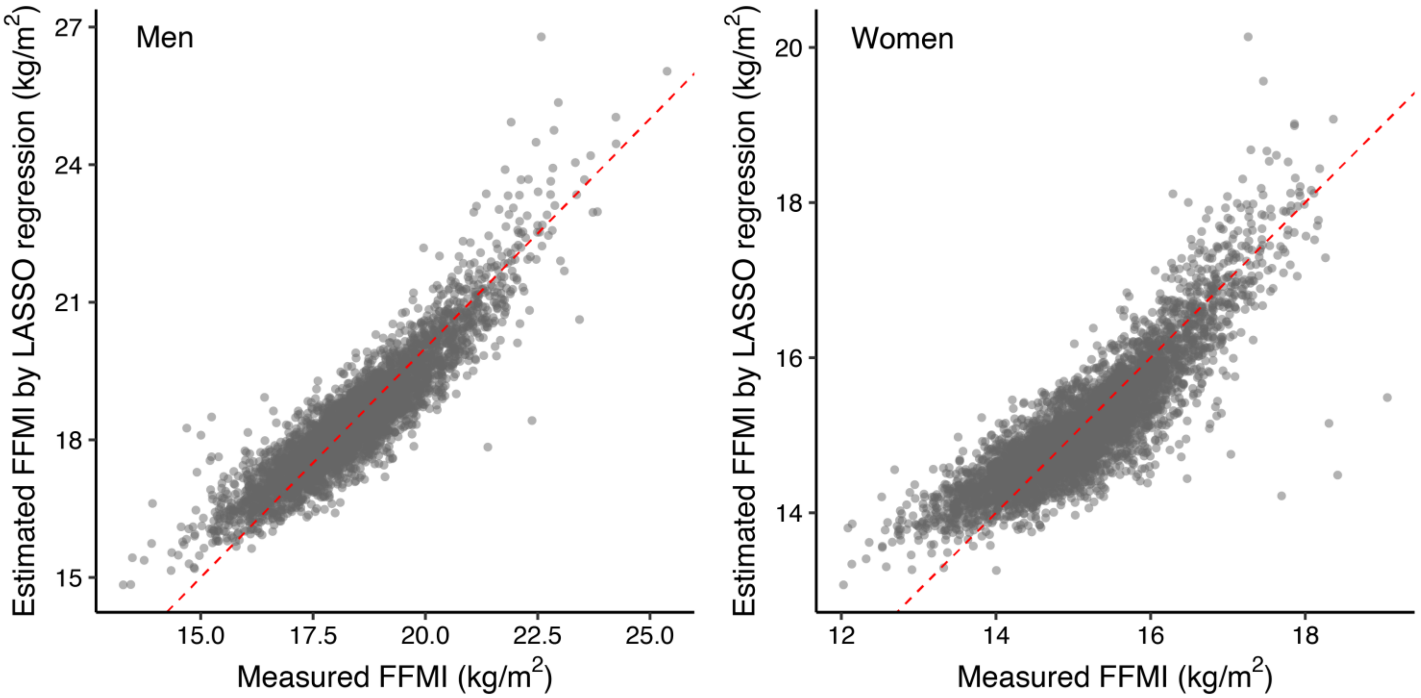

Figure S1. Correlation between measured and LASSO-estimated FFMI: Measured fat-free mass index (FFMI) was plotted against FFMI estimated by LASSO regression using BMI and eGFR in men **(left)** and women **(right)**. Each dot represents one participant, and the red dashed line indicates the identity line. The correlations between measured and estimated FFMI were 0.91 in men and 0.87 in women, respectively.

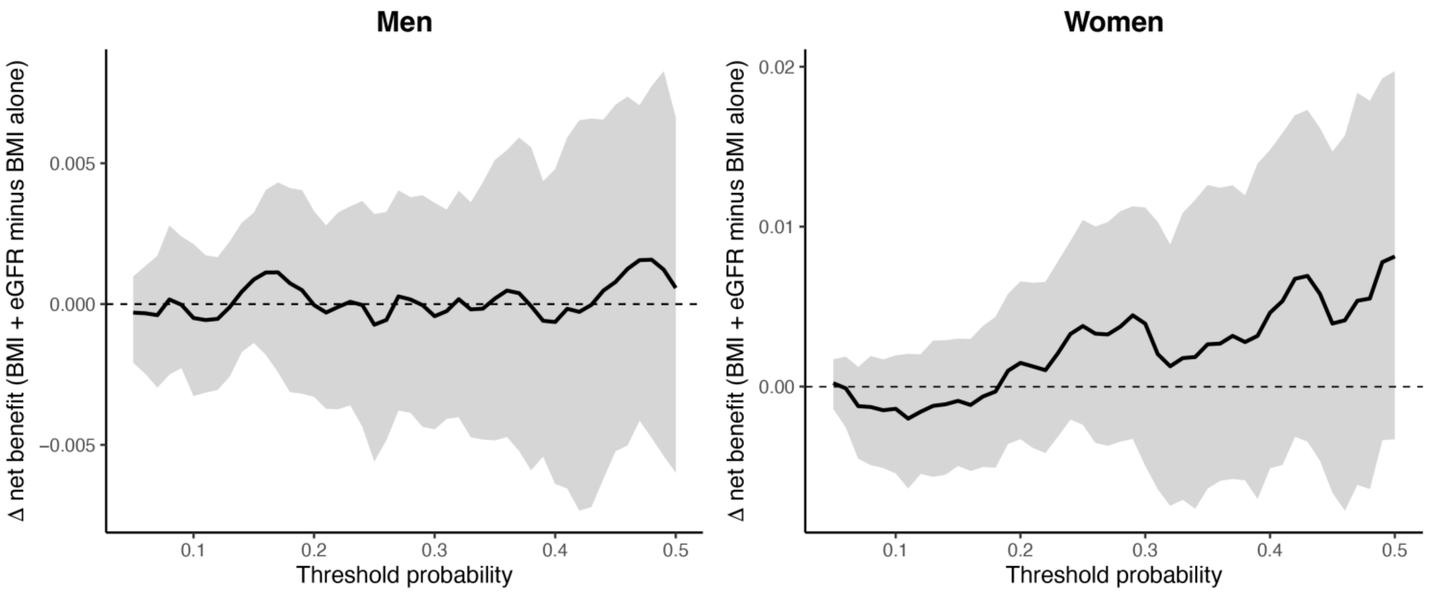

Figure S2. Incremental net benefit of adding eGFR to the BMI-only model. Incremental net benefit was calculated as the net benefit of the BMI–eGFR model minus that of the BMI-only model at each threshold probability using held-out test-set predictions from 500 repeated train–test splits. The solid line represents the mean incremental net benefit, and the shaded area represents the 2.5th–97.5th percentile empirical interval across repetitions. The dashed horizontal line at zero indicates no difference in net benefit between the two models. BMI, body mass index; eGFR, estimated glomerular filtration rate.

Table S1. Variable-specific missingness in the initially identified cohort

Domain	Variable	Available n	Missing n	Missing percent (%)
Demographics and body composition	Age	21265	0	0
	Height	21265	0	0
	Weight	21265	0	0
	Body mass index (BMI)	21265	0	0
	Fat-free mass index (FFMI)	19836	1429	6.7
	Waist circumference (WC)	19780	1485	7
Blood pressure	Systolic blood pressure (SBP)	21179	86	0.4
	Diastolic blood pressure (DBP)	21179	86	0.4
Hematology	White blood cell count (WBC)	19058	2207	10.4
	Red blood cell count (RBC)	21061	204	1
	Hemoglobin (Hb)	21061	204	1
	Hematocrit (Ht)	19108	2157	10.1
	Platelet count (PLT)	12966	8299	39
Glucose and lipid metabolism	Fasting blood glucose (FBS)	19832	1433	6.7
	Hemoglobin A1c (HbA1c)	17726	3539	16.6
	Total cholesterol (T-Cho)	21252	13	0.1
	HDL cholesterol (HDL-C)	21252	13	0.1
	LDL cholesterol (LDL-C)	21111	154	0.7
	Triglycerides (TG)	21252	13	0.1
	Cholinesterase (ChE)	8928	12337	58
	Uric acid	19007	2258	10.6
Liver function and protein	Total protein (TP)	10357	10908	51.3
	Serum albumin (Alb)	9722	11543	54.3
	Total bilirubin (Bil)	9514	11751	55.3
	Aspartate aminotransferase (AST)	21199	66	0.3
	Alanine aminotransferase (ALT)	21199	66	0.3
	Gamma-glutamyltransferase (γ-GTP)	21199	66	0.3
	Alkaline phosphatase (ALP)	15768	5497	25.8
	Lactate dehydrogenase (LDH)	9832	11433	53.8
Renal function and inflammation	Estimated glomerular filtration rate (eGFR)	17680	3585	16.9
	C-reactive protein (CRP)	9033	12232	57.5

The total initially identified cohort comprised 21265 participants. Missing percentage was calculated as the number of missing observations divided by 21265.

Table S2. Comparison of participants included in and excluded from the complete-case cohort

Variable	Included mean ± SD	Excluded available n	Excluded mean ± SD	Absolute SMD
Age	54.17 ± 11.77	12,529	45.38 ± 14.86	0.656
Height	163.89 ± 8.73	12,529	162.88 ± 8.38	0.119
Weight	61.20 ± 12.70	12,529	59.94 ± 13.03	0.098
Body mass index (BMI)	22.65 ± 3.62	12,529	22.47 ± 3.88	0.049
Fat-free mass index (FFMI)	16.70 ± 2.04	11,100	16.42 ± 2.05	0.137
Waist circumference (WC)	81.48 ± 10.06	11,044	80.62 ± 10.79	0.083
Systolic blood pressure (SBP)	119.47 ± 17.19	12,443	117.09 ± 17.26	0.138
Diastolic blood pressure (DBP)	75.59 ± 12.44	12,443	72.97 ± 12.99	0.206
White blood cell count (WBC)	5.30 ± 1.54	10,322	5.51 ± 1.51	0.137
Red blood cell count (RBC)	461.01 ± 44.47	12,325	461.20 ± 45.74	0.004
Hemoglobin (Hb)	13.94 ± 1.41	12,325	13.79 ± 1.46	0.105
Hematocrit (Ht)	42.17 ± 3.76	10,372	41.78 ± 3.88	0.100
Platelet count (PLT)	25.06 ± 5.93	4,230	25.82 ± 5.68	0.131
Fasting blood glucose (FBS)	92.85 ± 14.96	11,096	90.64 ± 17.47	0.136
Hemoglobin A1c (HbA1c)	5.72 ± 0.51	8,990	5.71 ± 0.58	0.029
Total cholesterol (T-Cho)	211.56 ± 34.39	12,516	205.48 ± 36.27	0.172
HDL cholesterol (HDL-C)	71.20 ± 18.91	12,516	70.70 ± 18.37	0.027
LDL cholesterol (LDL-C)	121.11 ± 30.15	12,375	116.68 ± 31.52	0.144
Triglycerides (TG)	96.26 ± 54.66	12,516	93.06 ± 80.72	0.046
Cholinesterase (ChE)	332.67 ± 72.94	192	347.73 ± 81.87	0.194
Uric acid	5.34 ± 1.34	10,271	5.28 ± 1.40	0.047
Total protein (TP)	7.19 ± 0.37	1,621	7.19 ± 0.37	0.003
Serum albumin (Alb)	4.34 ± 0.25	986	4.32 ± 0.26	0.064
Total bilirubin (Bil)	0.84 ± 0.34	778	0.82 ± 0.35	0.058
Aspartate aminotransferase (AST)	22.65 ± 9.01	12,463	22.05 ± 11.36	0.059
Alanine aminotransferase (ALT)	21.57 ± 15.22	12,463	20.93 ± 16.73	0.041
Gamma-glutamyltransferase (γ-GTP)	33.58 ± 39.70	12,463	30.66 ± 41.52	0.072
Alkaline phosphatase (ALP)	66.40 ± 19.20	7,032	66.72 ± 20.54	0.016
Lactate dehydrogenase (LDH)	170.64 ± 30.12	1,096	171.45 ± 31.61	0.026
Estimated glomerular filtration rate (eGFR)	71.85 ± 13.54	8,944	73.54 ± 13.73	0.124
C-reactive protein (CRP)	0.10 ± 0.30	297	0.11 ± 0.19	0.053

Values are presented as mean ± standard deviation (SD) using available observations for each variable. The included group represents the complete-case cohort (n=8736), whereas the excluded group comprises participants excluded because of missing data in at least one analysis variable (n=12529). “Excluded available n” indicates the number of excluded participants with available data for each variable. SMD, standardized mean difference.

Table S3. Sensitivity analysis comparing the BMI–eGFR model with the BMI–age–creatinine model

Sex	Model	N	AIC	Mean AUC (empirical interval)	Mean Brier score (empirical interval)	Calibration intercept (empirical interval)	Calibration slope (empirical interval)
Men	BMI-eGFR	4172	2364	0.946 (0.933–0.957)	0.0918 (0.0815–0.1033)	0.0079 (−0.2172–0.2114)	1.005 (0.858–1.161)
	BMI-age-Cr	4172	2420	0.948 (0.936–0.960)	0.0893 (0.0786–0.1009)	0.0071 (−0.2264–0.2194)	0.998 (0.843–1.166)
Women	BMI-eGFR	4564	3271	0.918 (0.902–0.933)	0.1149 (0.1034–0.1268)	−0.0007 (−0.1568–0.1442)	1.010 (0.880–1.167)
	BMI-age-Cr	4564	3276	0.919 (0.903–0.933)	0.1146 (0.1035–0.1264)	−0.0004 (−0.1581–0.1445)	1.009 (0.878–1.165)

AUC, Brier score, calibration intercept, and calibration slope are presented as the mean and 2.5th–97.5th percentile empirical interval across 500 repeated stratified 8:2 train–test splits. AIC was calculated from models fitted to the complete-case cohort. The empirical intervals are descriptive and should not be interpreted as conventional confidence intervals based on independent observations. AIC, Akaike information criterion; AUC, area under the receiver operating characteristic curve; BMI, body mass index; eGFR, estimated glomerular filtration rate; Cr, creatinine.

Table S4. Sensitivity analysis in the expanded model-available cohort

Sex	Cohort	N	Mean AUC (empirical interval)	Mean Brier score (empirical interval)	Calibration intercept (empirical interval)	Calibration slope (empirical interval)
Men	Original complete-case	4,172	0.946 (0.933–0.957)	0.0918 (0.0815–0.1033)	0.0079 (−0.2172–0.2114)	1.005 (0.858–1.161)
	Expanded model-available	7,647	0.951 (0.942–0.960)	0.0866 (0.0783–0.0952)	−0.0002 (−0.1374–0.1481)	1.005 (0.893–1.141)
Women	Original complete-case	4,564	0.918 (0.902–0.933)	0.1149 (0.1034–0.1268)	−0.0007 (−0.1568–0.1442)	1.010 (0.880–1.167)
	Expanded model-available	10,026	0.922 (0.912–0.931)	0.1117 (0.1048–0.1193)	−0.0010 (−0.0992–0.1027)	1.014 (0.923–1.123)

Values are presented as the mean and 2.5th–97.5th percentile empirical interval across 500 repeated stratified 8:2 train–test splits. The expanded model-available cohort included all participants with available FFMI, BMI, and eGFR, irrespective of missingness in other candidate variables. The empirical intervals are descriptive and should not be interpreted as conventional confidence intervals based on independent observations. AUC, area under the receiver operating characteristic curve; BMI, body mass index; eGFR, estimated glomerular filtration rate; FFMI, fat-free mass index.

Table S5. Internally validated performance of the BMI–eGFR model

Sex	N	Performance metric	Estimate (2.5th–97.5th percentile empirical interval)
Men	4172	AUC	0.946 (0.933–0.957)
		Youden cut-off	0.436 (0.377–0.473)
		Sensitivity	0.861 (0.821–0.899)
		Specificity	0.875 (0.836–0.904)
		Brier score	0.0918 (0.0815–0.1033)
		Calibration intercept	0.008 (−0.217–0.211)
		Calibration slope	1.005 (0.858–1.161)
Women	4564	AUC	0.918 (0.902–0.933)
		Youden cut-off	0.450 (0.408–0.487)
		Sensitivity	0.869 (0.831–0.905)
		Specificity	0.805 (0.765–0.845)
		Brier score	0.1149 (0.1034–0.1268)
		Calibration intercept	−0.001 (−0.157–0.144)
		Calibration slope	1.010 (0.880–1.167)

Values are presented as the mean and 2.5th–97.5th percentile empirical interval across 500 repeated train–test splits, except for the Youden cut-off, which is presented as the median and corresponding empirical interval. These intervals are descriptive empirical intervals and should not be interpreted as conventional confidence intervals based on independent observations.

Table S6. Complete sex-specific logistic regression results for the final BMI–eGFR model

Sex	Predictor	Coefficient	Standard error	Odds ratio	95% CI for OR	P value
Men	Intercept	29.9785	1.0514	1.046×10^{13}	1.332×10^{12}–8.213×10^{13}	<0.001
	BMI	−1.3639	0.0451	0.2557	0.2340–0.2793	<0.001
	eGFR	0.0112	0.0040	1.0113	1.0034–1.0192	0.005
Women	Intercept	18.1993	0.6275	8.014×10^{7}	2.343×10^{7}–2.742×10^{8}	<0.001
	BMI	−0.9781	0.0298	0.3760	0.3547–0.3986	<0.001
	eGFR	0.0312	0.0034	1.0317	1.0249–1.0385	<0.001

The final sex-specific logistic regression models were fitted using the entire complete-case cohort (men, n=4,172; women, n=4,564) to obtain the final model coefficients. Odds ratios for BMI and eGFR correspond to a one-unit increase in kg/m² and mL/min/1.73 m², respectively. BMI, body mass index; eGFR, estimated glomerular filtration rate; OR, odds ratio; CI, confidence interval.
